# A Qualitative Research on Usage Intention and Platform Swinging Behavior of Anonymous Social Applications “Soul”

**DOI:** 10.3390/bs12070230

**Published:** 2022-07-13

**Authors:** Xiaoxiao Song, Zhiyuan Yu

**Affiliations:** School of Journalism and Communication, Shandong University, Jinan 250100, China; songxiaoxiao@mail.sdu.edu.cn

**Keywords:** anonymous social media, Soul App, usage behavior, platform swinging, online communication

## Abstract

By iheriting online natural properties, anonymous social media (ASM) applications have become popular and have attracted large amounts of mobile users (e.g., the youth) who can construct new identities for role-play and show themselves in anonymous ways. In order to investigate the influencing factors toward usage intention (UI) and platform swinging (PS) behavior among anonymous social applications, we choose one of the most active ASM App “Soul” as the example in China and then conducte a semi-structured interview with 23 valid Soul users using qualitative methods. The results show that the factors, i.e., perceived usefulness, perceived ease of use, perceived privacy riskiness, perceived anonymity, subjective norms, emotional attachments, and perceived interactivity, indeed affect UI among Soul users via online communication. Moreover, we find that PS behavior is ubiquitous among interviewees and mainly depends on diversified needs, which consist of nine dimensions including different position and function among apps, usage comparison, friend migration, etc. Nearly 80% of interviewees believe that there exists a relationship between UI and PS, which can be described as a inverted U-shaped curve, i.e., the higher or lower UI, the less probability of PS. For the individuals’ social media usage behavior, a closed loop “Attitude–Intention–Behavior” is summarized. By conducting qualitative research, we intend to provide some insights and deepen the understanding of UI among ASM users in daily life.

## 1. Introduction

Anonymity is a natural property of the Internet at the moment of its birth, which caters to the psychological needs of users (e.g., voyeurism [1], attachments [2], self disclosure [3], social desirability [4], entertainment [5] and companionship [6]) and leads to the explosive growth of users on Bulletin Board Systems (BBSs), social networking sites (SNSs) and mobile applications from Web 1.0 to Web 2.0. Supported by the digital transformation in Web 2.0, a series of social media platforms/products has been operated for the purpose of interpersonal communication, such as Facebook, Twitter, Sina Weibo and Wechat in China. People can set up their user name and decide whether using their real-name or not voluntarily among the circle of friends or acquaintance. To some extent, real-name social networking deprives the users’ right of anonymous participation and may not meet diversified social demands. Therein, as a special category of social media, anonymous social media (ASM) applications represented by Soul, Momo and Tantan, etc., in China have opened up a new track in the era of mobile social media and simultaneously attracted large amounts of active users.

Soul App is regarded as a representative product of ASM in China, for which its accumulated number of users has exceeded 100 million. In March 2021, the average daily active users (DAUs) of Soul App had reached 9.1 million compared to that of 3.3 million and 5.9 million in 2019 and 2020, respectively. Therein, 73.9% of the DAUs born in or after the year of 1990 [7,8] are native to mobile Internet in China. Soul App is created as a virtual social “playground”, where Soulers can live in the “moment” to express themselves and share daily lives. With the characteristics of appearance-agnostic, interest-driven, decentralized, gamified, and AI-empowered, Soul is considered as the closest mobile App in the era of the metaverse [9]. For example, group chat parties and pet planet bring more diverse, interesting and rich social experiences for Gen Z users. The function called “Nielian” lets Soulers spontaneously create personalized avatars as their unique identity, which removes realistic distractions and allows users to express their preferences (or opinions) without pressure. Through the virtual space, the spiritual needs and senses of immersion can be satisfied.

### 1.1. Qualitative Research in Social Media Usage

Currently, social media has become an inseparable part in daily life, including activities for entertainment, learning and working. By qualitative methods, the usage studies of social media have been conducted in the research fields of marketing [10], health communication [11], pedagogy [12] and behavioral research [13].

For users’ purchase intentions, research studies in [14,15,16] found that the usage of social media can affect their intentions to purchase certain products or services. Gruzd et al. interviewed 51 scholars to explore the reason and to what extent scholars used social media for information exchange and dissertation [17]. For the Generation Z group, Liu et al. focused on the usage intention of social media during the COVID-19 epidemic and found that social media overload reinforced social fatigue and fear, which led to a decrease in the intention to continue the usage of social media [18]. Tsai et al. adopted in-depth interviews among adolescents to study Internet addiction and found that interviewees exhibited most of the symptoms of Internet addiction, e.g., compulsive use and withdrawal [19]. Nova et al. conducted a mixed-method research on ASM in Bangladesh via anonymous online survey and semi-structured interviews. The results showed that there exists a wide prevalence of sexual harassment on anonymous social networks and the lack of support for the victims [20]. Sharon et al. conducted in-depth interviews among ASM App–Secret users and found that anonymous communication in Secret was quite different from earlier forms of online anonymity, which included the degree of perceived anonymity and the sociotechnical arrangements that are likely to prompt deanonymization efforts [21]. Kang et al. explored usage intention, perception and interaction for anonymous App based on semi-structured interviews. Results showed that obtaining social validation from others was an important driver for participation and posting. Anonymous apps allowed more honesty, openness and diversity of opinion compared with others apps [22].

### 1.2. Cyber Migration and Platform Swinging

Since Web 2.0 was introduced, online SNSs became popular. For Internet users, it is common to be attracted by emerging applications and then jump to other sites or platforms in cyberspace.

Keaveney et al. [23] and Chen et al. [24] introduced consumer switching behavior from the market perspective to online platforms, which were considered as the embryonic stage of online user switching behavior. Cheng et al. proposed the phenomenon of network migration behavior in the usage of SNSs, which was defined as the behavior of users who mainly participate in using new social platform to replace the previous one [25]. It can be named as migration in the cyberspace. Xiao et al. viewed network migration as the behavior of users switching from SNS to the others [26]. Boyle et al. classified network migration behavior as place migration (i.e., never-return) and attention migration (i.e., jumping back and forth) [27].

In cyber migration studies, the Push-Pull-Mooring (PPM) model comes from the modern migration theory that is widely used [28,29], which is the most important theoretical contribution in the field of migration to date [30]. After introducing PPM, the studies of Internet usage behavior tend to be more conceptualized. Online migration research studies focus on blogs [31], SNSs [32], and traditional social media platforms [25,33] (such as Facebook, Twitter, Instagram, etc.). Hsieh et al. [34] proposed the post-adoption switching behavior of blog users by using the PPM model, and the push factors include weak connection and writing anxiety; pull factors include enjoyment and ease of use; mooring factors include switching cost (weak connection) and writing anxiety. Choi et al. enriched the concept of network migration by studying brand switching behaviors when shopping online and found that pull factors were the most influential on users’ switching [35]. Xiao et al. via the PPM model found that the factors using/maintaining inconvenience (anchoring) and acquiring new contacts (push-pull) influence users’ cyber migration behavior more than the factors of attractiveness, dissatisfaction and switch costs [26]. Hwang et al. applied social fatigue to social platform switching study, which included social overload (SNS Interaction overload), unwanted relationships, privacy concerns, etc. It was revealed that social fatigue was indeed a major factor for usage behavior [36].

With the emerging ASM and the change of target users for Y or Z generations, dating- or ASM-related studies have attracted more attention from scholars. The migration theory is introduced to study the user migration behavior from traditional social media platforms (or apps) to ASM apps. Based on migration theory and PPM, Gerhart et al. constructed a model to explore how anonymity influenced user behavior and found that users do not fully abandon traditional SNSs [37].

Platform swinging (PS) is a polysemous term with different meanings in the fields of mechanical engineering and medicine. In communication study, PS was proposed by Tandoc et al. based on the theory of polymedia, which describe the periodical rotation usage behavior among different social platforms. The significance of the swinging concept is that users’ needs are not only satisfied by the usage of social media but also by swinging between various social media, i.e., it does not mean that simply switching from one platform to another results in abandoning another. The users will maintain diverse types of social relationships via different social media platforms [38]. Actually, we can see that the foundation of PS is rooted in anthropology and sociology [39,40,41] and can be traced back to the study of migration in academic fields.

In this way, social media users can adopt malleable strategy in line with their demands by swinging among different apps or platforms. Tandoc et al. conducted a qualitative study to investigate platform swinging behavior among SNS users and found the users’ motivation for swinging, i.e., interpersonal management and self-presentation [38]. Liao et al. conducted a quantitative study to examine how factors (e.g., information value) influence consumers’ swinging behavior and cultivates brand loyalty. It was found that information value, social interaction value and self-presentation value had significantly positive impacts on consumers’ brand community-swinging, which further fosters brand loyalty [42]. Bolye et al. examined the accuracy of self-reported computer-specific time on multiple social media platforms and evaluated the influencing factors of reported accuracy based on platform swinging context [43]. Zhang et al. investigated how Chinese journalists navigate multiple social media platforms and employ data-driven source as complements to traditional source in practices during COVID-19. Platform-swinging was regarded as a variation impact the interviewees’ navigation of multiple sourcing options [44]. Larsson introduced PS into election campaigns by providing comparative insights of citizens on social media platforms ( including Facebook, Twitter, Instagram and YouTube) during 2017 Norwegian elections [45]. However, there exists few ASM or non-ASM works to study the relationship between UI and PS behaviors, especially among ASM users.

### 1.3. Research Question and Organization

In our prior work [46], we have combined the constructs of perceived anonymity (PA), perceived privacy riskiness (PPR), subjective norms (SN), emotional attachments (EA) and perceived interactivity (PI) with perceived usefulness (PU) and perceived ease of use (PEOU), which form an extended technology acceptance model to explore the UI of Soul App, which the youth account for a large proportion of users and the monthly active users reach 33.2 million [7,47]. It can be seen that the proposed model has relatively higher explanation for user intention (UI) among Soulers using quantitative methods. Based on our knowledge in ASM, we found that qualitative research on usage intention of specific ASM is quite limited. In this paper, we continue to conduct qualitative research by interview to further explore UI and then to create a supplementary method in addition to the quantitative method. The research questions (RQs) are presented as follows:

RQ1: How do factors influence Soul App UI?
RQ2: What are the factors eliciting PS behavior among Soul App users?
RQ3: What is the relationship between UI and PS behavior among Soul App users?

The organization of this paper is listed as follows: Section 2 introduces the qualitative analysis method including interview guideline and sample collection methods. Section 3 presents the preliminary analysis including coding procedures and basic data analysis. Section 4 discusses the main findings and summarize insights from the analytical results. Finally, Section 5 concludes this paper.

## 2. Research Method

According to the grounded theory [48], we analyze the collected textual data via qualitative analysis software NVivo11, which is commonly used for content analysis (such as interviews, open-ended questionnaires, etc.) and fully met the needs of analysis and coding for the grounded theory. In order to explore the usage intention and PS behavior, we conducted semi-structure interviews and then collected textual data from Soul App users.

### 2.1. Interview Guideline

By integrating previous research literature and combining participatory observations of the Soul App, we have compiled an outline of interviews about usage intention and platform swinging behaviors, which mainly includes the following: (1) demographic information (such as gender, age, occupation and income of the interviewees); (2) usage situations for ASM App; (3) the reason and UI to use ASM App; (4) situation of PS behaviors; (5) the relationship between UI and PS. The outline of the interviews is listed in Appendix A.

### 2.2. Sample Collection

We recruited a total of 25 Soul App users (i.e., Soul App and WeChat) to participate in in-depth interviews during the period of 20 June 2021 to 12 October 2021. Actually, due to the anonymity of Soul App, it was difficult to learn who the Soul user was. We can only recruit participants with two approaches: (1) online recruitment from Soul App, e.g., posting recruit advertisements in the Soul community or encountering the stranger by algorithm-driven matching; (2) offline recruitment, e.g., posting announcements to find nearby users. Finally, we recruited 21 interviewees online (20 were randomly recruited in Chat Party and 1 from community on Soul). Using the offline path, we recruited 4 interviewees from our university. The interview was conducting in Chinese Mandarin. Due to the geographic limitation, telephone interviews were in the majority and were supplemented with e-mail or face-to-face interviews. On average, the interview duration was 21 min for each interviewee. After the interviews, we promptly collated the recorded (or written material) to form interview data. Finally, the data from 23 valid interviewees were obtained, demographic information of the interviewees is shown in Table 1. Informed consent was obtained before the interview from all interviewees to ensure anonymity and to know the purpose of the research. We can see that the interviewees were born across four generations: post-1985s, post-1990s, post-1995s and post-2000s, with a male to female ratio of nearly 1:1. Moreover, all participants came from nearly 20 different cities in China and abroad, and students account for 52.17%.

## 3. Coding and Data Analysis

We organized all the audio and text materials collected from the interviewee and imported them into NVivo11. In order to understand the overview of all materials, we conducted a preliminary text analysis in terms of word frequency and present the core themes of the materials. As shown in Table 2, the top ten words in terms of frequency of occurrence are “usage”, “social”, “app”, “anonymous”, “swing”, “Soul”, “platform”, “influence”, “behavior” and “intention”. Therein, the frequency of top four words exceed more than 500.

**Figure 1:**
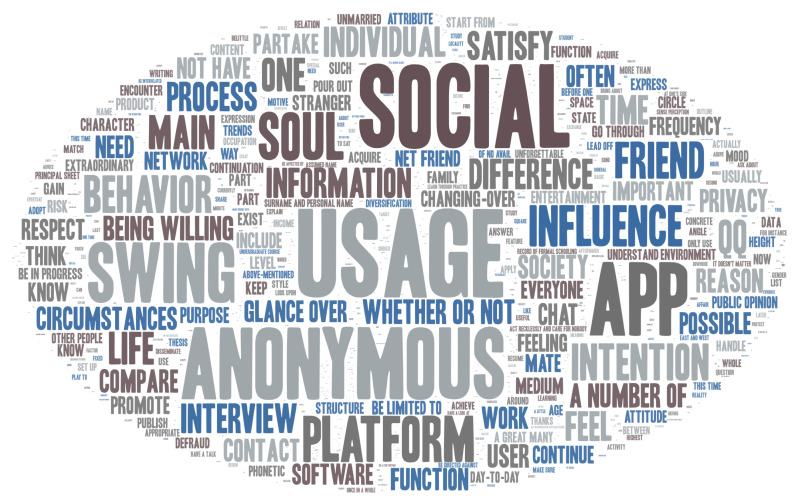
Word Cloud Map.

As depicted in Figure 1, the word cloud map more intuitively presents the scope and topics covered by the material content, with high-frequency words such as “usage”, “social”, “anonymous”, “app” and “swing” holding up the framework of the entire cloud map. After completing the basic word frequency query of the materials, we analyzed the 23 valid samples obtained by manual coding as the main method and automatic coding as the supplementary method for three levels of coding. The coding steps and results are as follows.

Open coding is the process of breaking down, chewing, labeling and redefining the material, which is the first step in conceptualizing and categorizing the original material [48]. In NVivo11, we coded the original material word by word. After completing the open coding, we categorized 236 nodes, explored the deep relationships within the nodes, merged semantically similar or related concept nodes into class genera and completed the axial coding, respectively. Finally, 12 classes of genera were obtained. Selective coding is the most crucial step in acquiring the core categories. Through repeated readings of the original data, we refined the 12 categories obtained by axial coding into six categories: motivation, prospection, perception, intention to use, platform swinging and use behavior. The coding results are shown in Table 3.

## 4. Discussion

After coding, we extract six categories (including usage motivation, prospection, perception, intention to use, platform swinging and usage behavior) from the original material and then explore the influencing factors and swinging behaviors.

### 4.1. RQ1 Influencing Factors toward Usage Intention: A Qualitative Complement

In our previous study [46], we have proposed the extended technology acceptance model and show that all hypothesis are valid via direct and indirect ways. In this part, we further conducted qualitative research and summarized the main topics as listed in Table 4 to answer RQ1.

In terms of PU, the interviewees would like to continually use Soul once their PUs are satisfied. For example, getting help, expanding horizons, making friends and improving mind during the period of usage are involved.

“*I met people from all walks of life, from whom I could obtain advice (or helps) for my work… When I had difficulties in writing my dissertation, I consulted my friends on Soul.*” (S3)

“*The impact on my life is that Soul App helps me regulate my emotions. I feel much better after venting.*” (S8)

“*I’ve made a lot of honest friends on Soul App, whom I’ve known for quite a long time and always kept in touch with.*” (S9)

Although usage frequency varies greatly, more than 90% of interviewees said they would use Soul for a long time because of the diverse functions to meet their needs and did not have better alternatives. Among the interviewed users, we can see that the stronger user’s perceived usefulness, the stronger the usage intention of Soul App.

In terms of PEOU and PI, ASM users would like to consider “whether it is easy to use” and “whether it is good to use”. Therefore, understanding PEOU is inextricably linked to PI. From the interviews, participants made a total of 32 statements regarding PEOU. Eighteen interviewees believe that Soul App is simple and easy to use.

On the one hand, the higher the level of PI, the stronger the UI to use. The user with higher usage intention think that Soul has a simple interface. On the other hand, five interviewees with lower usage intentions believe that Soul system performance needs to be optimized and too many functions seem redundant, etc.

“*(What attracts me is) the simple and fresh user interface. Soul App can provide the function of matching peers with same interest and the non-cheesy content on Square.*” (S23)

“*The system still needs to be optimized. At least no delay when sending pictures! No flash back! Otherwise, it affects the quality of user experience negatively.*” (S7)

For PPR and PA, participants made a total of 33 statements regarding the influence of PPR on UI and a total of 32 statements regarding PA on UI. Thirteen interviewees are attracted to using Soul App due to anonymity. More than half of them believe that anonymity can protect privacy and reduce risk concerns, which contributes to improving user experience.

“*In my opinion, anonymity can still play a role in protecting privacy.*” (S4)

“*I am willing to use Soul because I think it does a pretty good job in protecting privacy.*” (S2)

Moreover, users are not surprised that personal information and privacy on social media has the risk of leakage (e.g., Cambridge Analytica scandal in 2018). In this manner, most interviewees agree that there is no privacy on the Internet.

“*In the virtual world, we still need to be vigilant. After all, there are very odd mix of people.*” (S18)

“*Currently, I think we no longer have privacy... not much care about it.*” (S8)

However, when users perceive that the risk of online privacy is within control, UI will increase. When individuals are aware of privacy risk, the adoption of Soul App will decrease. In particular, this effect is magnified in ASM platforms. This explains the negative effect of PA on PR, i.e., the higher the level of PA, the lower the level of PPR [49]. At the same time, users also expect telecom operators or service providers to work together for privacy protection.

“*Be reasonable and not to expose privacy information, we can probably avoid this risk.*” (S15)

“*Anonymity is a better way to protect personal privacy. Like information on Sina Weibo ⋯⋯ can be easily collected. But if one is anonymous, the risk is relative lower. Because of that I am willing to use Soul App continuously.*” (S9)

“*Anonymity can protect personal privacy to some extent. ⋯⋯ Something the operators and service providers should do further work to protect our privacy.*” (S20)

In terms of SN, the majority of participants (*n* = 19; 82.6%) reported that the influence of SN from circle of friends has a significant effect on their PU. On the one hand, SNs are the users’ reception and reflection on the attitudes of important people around them. Accepting the influence of people around them will provide ideas for solving problems and will enhance the relationship among the close friends. On the other hand, the users connections established on ASM platforms are mostly weak compared with other apps used for acquaintances with strong connections. The following material reflects the interviewees’ attitude towards Soul in terms of SN.

“*Initially, my friend introduced Soul to me ⋯⋯ I found English corner is useful ⋯⋯ I think it help me to keep my study.*” (S4)

“*I will also recommend Soul App to friends. Legitimate use is not afraid of letting others know.*” (S19)

“*I don’t want people to know that I’m playing Soul because I don’t want the people around me to see my personal emotions.*” (S15)

In terms of EA, users who perceive more warmth and companionship during the usage are more likely to use it. The EA mentioned by interviewees include tweeting, confiding, companionship, caring for others, tree hollows, etc. The time and space barriers of communication are broken via Internet, but people’s social circle becomes smaller and smaller [50]. Expanding social interaction, meeting interesting souls and gaining emotional recognition are vital experiences when using social media.

“*I met a very interesting person located in Tianjin. I would fall in love with him in reality world. We often chat and share all kinds of interesting things ⋯⋯⋯⋯ Also I met a depression patient and gradually learn the symptom. Via Soul, I care about him and so on.*” (S11)

“*When I first used Soul, I met a senior from another university who was in grad school. He is currently studying abroad for Ph.D. We followed each other WeChat and Sina Weibo accounts and still chatted or commented the posts occasionally on Soul during those two years.*” (S23)

“*I met my ex-boyfriend on Soul. Story of online love came true.*” (S10)

In summary, ASM Apps capture the main point that users seek solace but cannot obtain it. In virtual social environments, Soul encourages people to show themselves and meet strangers. When users obtain the satisfaction of EA through a series of usage, the continuing usage intentions will increase. Similarly, we can see that social validation, obtaining connection with people, sharing information are common intentions for using ASM compared with the studies in [21,22].

### 4.2. RQ2: Platform Swinging Satisfy Users’ Diversified Needs

In the interview, the participants’ PS behaviors can be divided into three categories according to the platforms types: swinging between ASM Apps, swinging between anonymous and non-anonymous Apps and swinging between non-anonymous Apps, which often co-exist. Among all participants, 22 had PS behavior when using social media, of which 15 participants were involved in all three types of swinging behaviors. 11 interviewees mainly swing between anonymous and non-anonymous platforms, and 14 participants used to experience swinging between different ASM Apps.

In this part, we explore factors and reasons towards PS behavior to answer RQ2. The wide variety of individual potential needs triggers different usage behaviors. Based on Internet use and gratification theory, those needs can be summarized as cognitive needs, affective needs, social integration needs and the needs of releasing tension, relieving fear of separation, stress and confusion [51,52,53,54,55]. By PS, users can consciously choose platforms in order to complete different tasks and meet different needs [56].

“*On Soul, I can meet people from various background and learn a lot from them. Moreover, I can improve pleasure in life and gain surprises or joy from them.*” (S3)

“*(On the App) I met a girl who was depressed and said she wanted to commit suicide, I talked to her for a long time and persuaded her nicely to give up the idea, I thought I was pretty great at that time.*” (S14)

“*For example, I didn’t know what ‘Mianji’(meet online friends) meant till using Soul, I think it will help to chase some craze among fashionable young people and trigger a bit of relatively new and novel behavior and habits.*” (S4)

“*WeChat was used to contact family, friends and deal with work; QQ is used as a supplement to contact previous classmates who didn’t use WeChat; and Soul is for passing time and entertainment.*” (S17)

“*When failing college entrance exam, I was very stressed and sleepless at night feeling no confidence to take the school exam again ⋯⋯ Via Soul, I matched with a guy by the function of voice calling ⋯⋯ As a strange senior friend, He gave me a lot of good advice to help me build the confidence. I really appreciate in that period of time (he) very patient to listen to my grievances and so on. Everyone around me was under a lot of pressure during that time, so (coming across him) indeed give me some confidence and stabilize my emotions.*” (S1)

It is worth mentioning that PS is related to the need of expanding (or maintaining) the circle of friend. According to the interview, more than half of the participants mentioned that expanding their friend circle is the reason for PS behavior. When the relationship between strangers becomes more intimate on ASM App, strong ties will be formed by following frequently used social media account, i.e., enter the social circle of acquaintances successfully. Meanwhile, users will reduce use frequency when they found the specific App lost novelty and interest.

“*To have a wider social circle and see something I haven’t done before.* (S7)

“*I’ve met some friends that I can continue to develop into reality, and they’re all very nice and helpful.*” (S10)

“*The usage between WeChat and Soul is actually complement for circles of acquaintances and circles of strangers, respectively.*” (S11)

“*I think the motivation of platform swinging is to realize the evolution of friend relationship. If you develop a good friend on Soul, you would like to keep more contact (e.g., following WeChat account each other.*” (S9)

After coding analysis, we found that the fundamental starting point and purpose of users’ PS behavior intend to satisfy the diversified needs by using various platforms rather than single Apps. Moreover, nine dimensions of “diversified needs” for PS are summarized, as shown in Table 5. Therein, the dimension in which different positioning and functions cannot be replaced is regarded as the most important one.

The usage allocation of platform mainly refers to that the time and energy spend on different platforms. Depending on the needs and purposes, the interviewees can decide which platform or App is used independently. For example, users usually contact family members or friends on WeChat and Tencent QQ, pour out their grievances, encounter strangers on ASM App (e.g., Soul and Summer), check current affairs news on media agency Apps or Sina Weibo, and look for learning materials and advice on Zhihu (Quora in China), etc.

“*QQ is mainly used for student work, school notifications. WeChat is to contact family and older people. Zhihu is used for searching answers. Soul is mainly to see other people’s moments and chat each other. Summer feels fresh at first because it is a campus community.*” (S2)

“*WeChat, QQ mainly for family chat, Facebook, instagram, Snapchat to share life and chat with family. Soul, summer, tinder, Tan Tan are used to find a girlfriend.*” (S21)

Compared with non-ASM applications (e.g., Facebook, Twitter, Snapchat) [38], we find that PS behaviors help users meet their diverse needs and fulfill their gratifications, e.g., relationship management, regardless of anonymous or not. As a conclusion, the interviewees’ PS behavior cover nine dimensions, which include different positioning and functions, usage comparison, platforms migration among friends, obtaining different experience, etc. According to different usage needs, users allocate their usage among diverse social platforms to form a personalized swing behavior.

### 4.3. RQ3: The Relationship between UI and PS

PS can satisfy the users’ diverse needs for self-presentation and relationship management [38]. However, the relationship between UI and PS is still unclear. Therefore, we further investigate users’ perceptions of the relationship between UI and PS to answer RQ3.

Based on functional attributes, the social platforms mentioned in the interviews are divided into two categories: non-anonymous acquaintance social Apps (e.g., WeChat, QQ, Facebook, etc.) and ASM App or online community with anonymity (e.g., Soul, Tinder, Summer, Goodnight, Clubhouse, Zhihu, Hupu, etc.). From the interview, the largest number of social platforms used by participants is 10 and that of the smallest is 2. The average number is around five platforms. Among all participants, 18 participants believe that there exists a link between UI and PS, and the level of UI regularly affects the probability of PS behavior.

S7 becomes a Souler nearly a year with high UI. He keeps contact with friends for a long time. However, Soul does not exert a major role in daily life. The needs of handling paperwork, contacting family members and friends, obtaining information, entertainment and relaxation allow him to swing between different social platforms. Interviewee S7 believes that there is an inevitable link between UI and PS.

“*I will frequently use a special App if I have more demand, which increase UI. For me, WeChat is usually used to contact colleagues, friends, and relatives. If you are not interested in an App, you certainly will not use it. So that the higher or lower UI will affect my usage behavior.*” (S7)

Interviewee S5 used to spend more than 1000 days on Soul, but cancelled Soul account now. In his opinion, traditional social platforms represented by WeChat are essential tools for daily life and work, while Soul is relatively dispensable. There is a link between UI and PS depending on personal differences.

“*It depends on the individual. For me, I can’t contact my friends and family without WeChat, but Soul has no such big influence on my life, i.e., it’s optional. If I don’t want to use Soul, I won’t use the similar Apps.*” (S5)

Interviewees S2 and S23, who are both college students and have more time to use various social platforms. They agree that it has a correspondence between UI and PS.

“*My current UI for ASM App is particularly low and I just post the status and view other followed users’ activities in a short time. PS behavior doesn’t come up very often.*” (S23)

“*If the UI is high, I will spend a lot of time to chat, which this app becomes my main battlefield. In this way, frequency of my swaying among different platform will be reduced. If UI is very low, I will use it for a while and then abandon it after the novelty wearing off quickly.*” (S2)

In summary, the higher or lower UI is, the lower the probability of PS behavior. The highest probability of PS behavior occurs when UI is kept at a medium state. In addition to UI, the interviewees also suggested several other factors that influence PS, including personal preferences, social fatigue, applied scenarios and usage motivation.

“*Generally, PS is related to individuals’ needs and characteristics. If a certain App can meet my social need, I don’t need to swing; Sometimes, even if I’m not very interested, I will occasionally log in a specific App considering my previous happy times on it.*” (S1)

“*Sometimes, social media fatigue also pushes me to other Apps to distract and get relaxed.*” (S20)

“*When UI is high, PS behavior between soul and WeChat occurs due to urgent contact from friends; or PS behavior between Soul and Weibo occurs when Soul is waiting for response.*” (S23)

“*The main functions of social platforms are different. I use WeChat, QQ, etc. to satisfy my diversified demands. So I will continue to swing. I think UI is one side, the motivation to use is the other side.*” (S13)

In addition, five participants believe that PS is not influenced by UI. They hold the point that PS has objective existence and will not be changed based on the degree of UI.

“*I think there is no link between UI and PS. For example, If I use Soul App now, I would like to swing another App recommended by my friends, which function works better than Soul. I have an inclusive attitude towards new things, and personally think the high or low UI has no influence on PS.*” (S4)

“*There exists difference between Soul and Clubhouse in term of English group chatting: feel difficult on clubhouse I will swing to soul to find confidence. (I think UI ) has no impact on PS, depending on whether the friend set up a chatting room or not.*” (S16)

“*I will do PS regardless of high or low UI. Due to the fact that one App can only target specific user group or certain topics, it is necessary to obtain more information from various platforms via PS behavior.*” (S22)

Regardless of the degree of UI, social media apps with diversified services can satisfy immediate needs and tend to be frequently used. Moreover, the diversified services provided by social apps with different positioning can better meet their usage needs without spending too much effort.

### 4.4. Implication and Limitation

**Figure 2:**
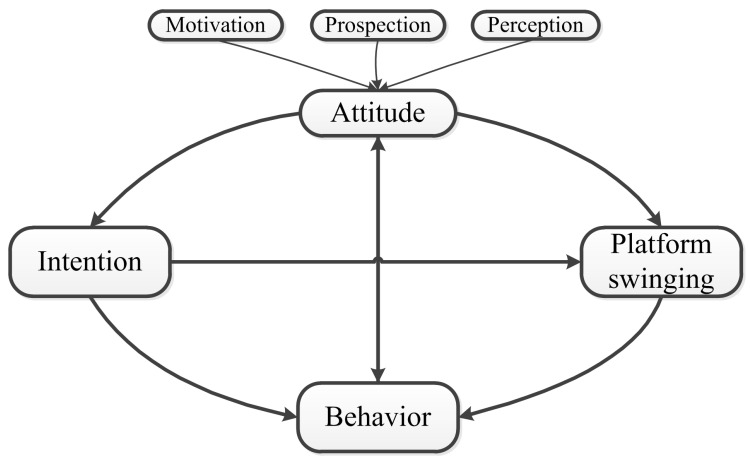
Category Relationship Diagram.

In term of the theoretical implication, we sorted out the relationships between the categories of attitude, UI, PS and user behaviors after data analysis and discussion. As shown in Figure 2, user behavior is the verification of usage prospection and the most critical step in the closed loop “Attitude–Intention–Behavior” among individuals’ social media usage. In cyber-psychology research, motivation is regarded as one of causes that trigger online usage behavior [57]. People can obtain online satisfaction from socializing, consuming, entertainment, etc. The stronger the satisfaction, the more online usage will be obtained [58]. When users’ prospections have been satisfied during the usage, their continuous UI will increase, The more useful, anonymous, emotional and interactive the user perceives it to be, the more positive the attitude will be and higher UI is obtained, which will in turn affect usage behavior. On the contrary, if the initial usage prospections are not satisfied, UI will be greatly reduced and then this will affect their subsequent usage behavior. By verifying usage prospection, user’s attitude towards certain product will fluctuate. As a trigger for the usage behavior, the fluctuation of attitude will become the starting point of a new cycle, which will influence the next usage behavior, forming a closed loop of “Attitude–Intention–Behavior–Attitude”.

In this study, there exists some limitations that are summarized as follows: First, lack of long-term observation of Soul App users. In the process of the interview, users’ responses can only reflect their perceptions at specific times and conditions. Therefore, the data of observing participants’ UI cannot reflect the long-term impact of variables (such as PU and PEOU) on UI to a certain extent. Second, the proportion of students (including college, undergraduate and graduate students) is relatively high among 23 valid interviewees. Third, the sample’s size can be further increased. In the further, other research methods (e.g., focus groups) can be introduced to enhance in-depth observations.

## 5. Conclusions

In this paper, we have conducted qualitative research by semi-structured interviews to explore influencing factors towards usage intention (UI) and platform swinging (PS) behavior among Soul users. The mutual relationship between UI and PS is also revealed. We have found that the factors of perceived usefulness (PU), perceived ease of use (PEOU), perceived privacy riskiness (PPR), perceived anonymity (PA), subjective norms (SN), emotional attachments (EA) and perceived interactivity (PI) would impact on UI, which can supplement the prior qualitative study. Therein, PEOU is inextricably linked to PI and users consider PEOU through “ease to use” and “good to use”; manageable privacy risks are acceptable for users and anonymity protects privacy in some extent; SN impacts UI indirectly and mainly resulted in personalized motivations; EA tended to be a vital factor influencing ASM users’ UI. In terms of PS behavior, we can see that satisfying diverse needs (e.g., different position and function, usage comparison, obtain different experience, etc.) exerts a vital role when users tend to swing among various platforms. Most interviewees believe that a certain connection between UI and PS exists, i.e., the higher or lower UI, the lower the probability of PS, which looks similar to an inverted U-shaped curve. In addition, personal preferences, social fatigue, applied scenarios and usage motivation also impact PS. By conducting this qualitative research, we aim to provide a meaningful perspective to guide the usage behaviors of anonymous social media.

For future studies, we will focus on anonymous social media users’ specific PS behavior and introduce relevant communication psychology theories to explore the behavior of users, e.g., self-presentation in PS process. In particular, as the number of valid interviewees increases, there will be more valuable factors revealed from the interview, which will contribute to enrich our findings.

## Figures and Tables

**Table 1:** Demographic Information.

	Variable	Num. of Participants	
Gender	Female	12	52.2%
	Male	11	47.8%
Age	18–25	17	73.9%
	26–30	4	17.4%
	31+	2	8.7%
Martial Status	Single	20	87.0%
	In love	3	13.0%
History of usage	Within 1 year	6	26.1%
	1–3 years	10	43.5%
	More than 3 years	7	30.4%
Frequency of use	every day	11	47.8%
	Once every few days	6	26.1%
	Once per week	4	17.4%
	Once every few weeks or less	2	8.7%
Usage time per login	Within 1 h	12	52.2%
	1–3 h	8	34.8%
	More than 3 h	3	13.0%

**Table 2:** Word Frequency Top 10 (in Chinese).

Word	Length	Count	Weighted Percentage (%)
usage	2	881	4.26
social	2	785	3.79
app	3	603	2.91
anonymous	2	579	2.80
swing	2	410	1.98
Soul	4	259	1.25
platform	2	240	1.16
influence	2	192	0.93
behavior	2	159	0.77
intention	2	154	0.74

**Table 3:** Coding Results.

Coding Procedure	Subtheme	Example of Relevant Quotes/Catalogues/Concepts
Open Coding	ASM app leads the social trend	“*I think Soul will lead to craze trend among fashionable youths and* *trigger some emerging behaviors and habits.*” (S4)
	ASM app affects emotional states	“*I met my girlfriend on Soul App and we recorded moments of life* *which is great impact on me.*” (S19)
	Curiosity	“*I saw Soul ads from QQ and downloaded to satisfy curiosity. I can* *meet different kinds of people and sense the world via Soul.*” (S7)
	Continuous usage intention	“*I intend to continually use. Because Soul is a nice to share some* *views, moods, opinions, etc., as a tree hole.*” (S9)
	Perception of privacy risk	“*Be careful, don’t post any message related to individual’s privacy.*” (S10)
	Reasons for platform swinging	“*Because the function is different. Depends on the social needs,* *sometimes keep in contact with acquaintances or strangers.* ” (S6)
	Relationship between swinging and intention	“*It has some connection and influence. With the higher intention,* *I may not use other apps with similar functions. But other app with* *complementary function is still used.*” (S20)
	Involvement in usage process	“*I often read the posts and browse the moments of other Soulers* *with higher involvement.*” (S12)
	Functions commonly used	“*I often match (or connect) with other Soulers to see their* *interesting moments on the square.* ” (S13)
Axial Coding	Individual effects	Relieving stress, getting information, satisfying the desire to share, tree holes, socializing with singles, etc.
	Social effects	Triggering trends, promoting market competition, lonely gathering places, cyber violence, underage issues, etc.
	External factors	Systematic interactivity, anonymity, riskiness
	Subjective feelings	Subjective norms, usefulness, emotional attachment, ease of use, etc.
	Purpose of use	Voyeurism, socialization, confession, recreation, etc.
	Reasons for use	Curiosity, entertainment, anonymity, friendship, etc.
	History of use	Contact time, use time, continuity of usage, etc.
	Usage process	Involvement, usage frequency, main functions, social platform choice, etc.
	Continuous use	Intentions to use for a long time, willing to return, unable to give up usage
	Stages of use	Intermittent use, uninstallation and reinstallation
	Migration of social apps	Group influence, work (study) needs, etc.
	Swing of social apps	Contrast use, swing and willingness, etc.
Selective Coding	Motivation	Reason for use, purpose of use
	Prospection	Individual effect, social effect
	Perception	External factors, subjective feelings
	Intention	Continuous use, stages of use
	Platform swinging	Migration of social apps, swinging among social apps
	Usage behavior	Use history, use process

**Table 4:** The reasons and purposes of usage intention.

Influencing Factors	Data Classification (Num. of Nodes)
Perceived usefulness	Getting help (3), entertainment (2), expanding horizons (2), meeting people (2), enhancing life pleasure (1), improving mindfulness (6), finding companionship (4)
Perceived ease of use	Convenient to use (23)
Perceived riskiness	Acceptable to certain risks (3), concerned about privacy agreements (1), no privacy on the Internet (6), operators should protect personal privacy (4), raise their own awareness of prevention (11)
Perceived anonymity	Attracted by anonymity (12), anonymity protects privacy (6), anonymity reduces concerns (4), anonymity has advantages and disadvantages (4), maintain a neutral attitude (2)
Subjective norms	Unwilling to let friends know (7), willing to not hide too much (7), willing to share part of it (2), indifferent (4)
Emotional attachments	Share spit and pour (10), touching and warm (3), care about depressed users (2), meet emotional needs (2), meet interesting people (10), tree holes (4), fall in love (4)
Perceived interactivity	Various functions (6), simple interface (1), interesting functions (1), system performance needs to be optimized (4), unclear situation of social environment (3)

**Table 5:** Reasons for platform swinging.

Reasons for PS (Num. of Nodes)	Corresponding Materials
Different positioning & functions (13)	*“Considering each App is with specific characteristics, I will adopt* *the proper one.”* (S2)
App/platform Comparison (3)	*“When I find other interesting apps, I may use them in comparison.”* (S11)
Platform migration among friends (2)	*“At first I only used QQ, but then I got more and more friends on WeChat* *and it us slowly instead of QQ.”* (S6)
Get different experience (3)	*“I can get different experiences among apps, so I choose to swing.”* (S3)
Group influence (1)	*“Because many good friends use this app, so I have to use it. I think it is* *the group influence.”* (S13)
Meet social needs (1)	*“I am a very social person. These apps allows me to socialize very* *well.”* (S1)
Context-specific usage (1)	*“The school sends out notices usually via QQ group-chatting, but I also* *need to use WeChat to contact friends and expose secrets on Soul.”* (S8)
Freshness (1)	*“By PS, I can keep to feel fresh.”* (S9)
Complementary to socializing with acquaintances (4)	*“I use Soul due to its anonymity. The circle of friends is more extensive* *and I can see the posts.”* (S7)

## Data Availability

Not applicable.

## Abbreviations

The following abbreviations are used in this manuscript:

ASM	Anonymous social media;
DAUs	Daily active users;
EA	Emotional attachments;
PU	Perceived usefulness;
PEOU	Perceived ease of use;
PA	Perceived anonymity;
PPR	Perceived privacy riskiness;
PPM	Push-pull-mooring;
PS	Platform Swinging;
PI	Perceived interactivity;
SN	Subjective norms;
SNSs	Social networking sites;
UI	User intention.

## Appendix A. Interview Outline

Part 1. Personal information

1. Your name (you may use a pseudonym or nickname)
2. Your gender
3. Your age
4. Your highest education
5. Your occupation
6. Your emotional state
7. Your income

Part 2. Usage of anonymous social apps

1. Which anonymous social apps are you using/have you used?
2. When did you start using these anonymous social apps?
3. How often do you use them (how often do you log in, how long do you use each time you log in)?
4. What specific features do you mainly use?
5. How involved are you in the process of using them (e.g., do you post often, view other users’ posts)?
6. Please tell us your opinion about anonymous social apps from the perspective of personal use and social influence (public opinion attributes).

Part 3. Motivations of UI for anonymous social apps

1. Why do you use anonymous social apps/what do you want to achieve?
2. What is the impact of using anonymous social apps on your work and life? (Is it useful to improve your life and work performance, and what role does it play)
3. Do you think it is important to maintain a fair amount of anonymity while using the app?
4. Would you like to let your friends/family know that you are using anonymous social apps? Why?
5. What do you think about the privacy risk of using anonymous social apps?
6. What are your memorable experiences in using anonymous social apps? (emotion, catharsis, hollow, etc.)
7. Would you like to continue using your current anonymous social app and why?
8. What settings, features, styles and other product characteristics do you think would enhance your willingness to use?

Part 4. Platform Swinging Behavior

(Platform swinging behavior of social media refers to users swinging and switching between two or more social media apps for social purposes, for example, I use QQ to contact others while using WeChat, and I often swing between the two.)

1. Do you have platform swinging behavior when using anonymous social apps?
2. Do you swing between different anonymous social apps (e.g., between Soul and Tan Tan) or between anonymous social apps and social apps (e.g., between Soul and WeChat)?
3. Please list the names of all the social media platforms (including but not limited to anonymous social media platforms) you use for swinging.
4. Please briefly describe the main social activities you do on each of these social platforms, i.e., how and why you use them. (For example, I mainly use WeChat, QQ, Soul and Tan Tan. In WeChat and QQ, I can get in touch with my family and friends, post my daily news, etc. In Soul and Tan Tan, I match strangers, browse my friends’ profiles, expand my social circle, etc.)
5. What needs do you meet with platform swinging? (e.g., to expand your circle, to get better information, to get entertainment, etc.)
6. For what reasons do you swing between different social apps (including but not limited to anonymous social apps)?
7. How does your willingness to use apps affect your platform swinging behavior? Why? (Do you continue to swing between platforms when your intention to use an anonymous social app is low/high? (Please explain why)
